# Demonstrating the Potential of a Low-Cost Soil Moisture Sensor Network

**DOI:** 10.3390/s22030987

**Published:** 2022-01-27

**Authors:** Ciprian Briciu-Burghina, Jiang Zhou, Muhammad Intizar Ali, Fiona Regan

**Affiliations:** 1 DCU Water Institute, School of Chemical Sciences, Dublin City University, Dublin 9, D09 E432 Dublin, Ireland; fiona.regan@dcu.ie; 2 School of Electronic Engineering, Dublin City University, Dublin 9, D09 E432 Dublin, Ireland; jiang.zhou@dcu.ie (J.Z.); ali.intizar@dcu.ie (M.I.A.)

**Keywords:** dielectric permittivity, LoRaWAN, TDR, soil moisture

## Abstract

Soil moisture is a key parameter of the climate system as it relates to plant transpiration and photosynthesis and impacts land–atmosphere interactions. Recent developments have seen an increasing number of electromagnetic sensors available commercially (EM) for soil volumetric water content (θ). Their use is constantly expanding, and they are becoming increasingly used for agricultural, ecological, and geotechnical applications and climate research, providing decision support and high-resolution data for models and machine-learning algorithms. In this study, a soil moisture sensor network consisting of 10 Sense Cap capacitance-based sensors is evaluated. Analytical performance of the sensors was determined based on laboratory and field measurements with dielectric permittivity (ε) standards and soil media substrates. Sensor response normalisation to standards of known ε was found to reduce intersensor variability and provide robust estimates of θ in soil samples with known θ. Cross-comparison with a time-domain reflectometry (TDR) instrument carried out in two soil media demonstrates good agreement between the two probes throughout the tested range. The data communication performance of the network was evaluated in terms of packet drop rate at different ranges and sampling frequencies. It was noticed that the drop rate increased with distance from the gateway, while sampling frequency had no effect. Sources of errors associated with probe installation were identified and recommendations are provided for sensor deployment. The off-the-shelf all-in-one solution provided by Sense Cap is low cost, user friendly and suitable for implementation at temporal and spatial scales once the identified shortcomings are addressed. The evaluation presented aims to aid stakeholders and users involved in soil and land management practices including crop production, soil conservation, carbon sequestration and pollutants transport.

## 1. Introduction

Soil moisture is an essential parameter for irrigation management, transport of pollutants and estimation of energy, heat and water balances [1]. Soil moisture is one of the most important soil spatial-temporal variables due the highly heterogeneous nature of soils which in turn drives water fluxes, evapotranspiration, air temperature, precipitation and soil erosion [2]. The capacity of land to act as a carbon sink critically depends on the nonlinear response of carbon fluxes to soil moisture and on land–atmosphere interactions [3]. Soil moisture can reduce primary production [4,5] and intensify climate extremes through land–atmosphere feedbacks [2]. Traditionally, a range of methods exist for measuring soil moisture and include thermogravimetric, neutron scattering and the use EM sensors [1]. Emerging techniques include ground-penetrating radar [6], the measurement of cosmic neutrons [7,8] and remote sensing [9]. Although the emerging techniques are attractive due to their spatial capabilities, they have limitations. For example, remote sensing only captures soil moisture from the top soil layers, provides large-scale estimates (km resolution) and does not resolve all the forms of land water storage. Ground-truth data remain crucial for the calibration and validation of remote sensing products [10]. Such data can be obtained using gravimetric sampling, which is a labour-intensive, destructive technique, or through the use of EM sensors which can provide continuous high-frequency data and spatial distribution when used in a network [11]. EM sensors respond to the soil dielectric permittivity (ε) which is used to determine θ. Time-domain reflectometry (TDR) and transmissometry (TDT) sensors operate in the GHz range, while the impedance and capacitance sensors operate at lower frequencies from 20–300 MHz [12,13]. In general, low-frequency sensors are much cheaper but more sensitive to cofounding effects of salinity, temperature, soil texture variability and imaginary dielectric permittivity [12,14]. Though the TDR-method-based sensors are regarded as the most accurate EM-based technique for θ, their use in distributed sensor networks is limited due to the high cost. Instead, low-cost sensor networks are being increasingly employed for climate research [11], irrigation management [15,16,17] and validation of spatial methods [10,18].

Soil moisture sensors operating within wireless sensor networks (WSNs) provide a significant reduction in wiring and harness and offer enhanced flexibility in network design and implementation, are easier to deploy and cost effective. Over the past few decades, a variety of wireless communication protocols have been designed and adopted for different applications. Each of these protocols are designed to meet specific requirements suitable for a particular set of applications. Mostly, wireless communication networks are evaluated based on their characteristics such as transmission range, data rate and power consumption. Wireless communication systems with high data rate (due to usage of higher frequency) can only support short-range transmission (due to limited wavelength). For example, Wi-Fi can support high data rate but can only support short-range data transmission. ZigBee and Bluetooth support a short range with low data rate designed for applications requiring point-to-point short-range data transmission. Cellular networks (2G to 5G) support long-range and high data rate transmission, but often struggle due to high power consumption and data transmission costs due to limited bandwidth and high-cost infrastructure. While each of these protocols are suitable for a certain set of applications, there is no one solution fit for all wireless communication protocols. For smart farming and environmental monitoring, a wide coverage over a large geographical area is often required and usually sensors must be battery-powered due to their remote location. LPWAN is a wireless technology with characteristics such as large geographical coverage, low bandwidth, and data rate suitable for sensor observations captured at long intervals and low power consumption, which make it suitable for environmental monitoring applications. LoRaWAN technology is currently one of the most promising protocols for wireless communication. This network is relatively easy to implement, has a ready-to-use security layer and a guarantee of wide coverage with minimal maintenance and low energy consumption, which is ideal for large-scale use [19]. LoRaWAN-enabled soil moisture sensors are commercially available, such as DecentLab (Dübendorf, Switzerland), AMIT Wireless (Tainan, Taiwan), Agro Sense (Budapest, Hungary), ICT International (Armidale, Australia), TekBox (Woodhurst, UK), SensoTerra (Utrecht, The Netherlands) and Seed Studio (Shenzhen, China). Among these suppliers, Seed Studio though their Sense Cap range provide a full solution, including gateway and data management at the lowest cost on the market (sensor node—EUR 185, gateway—EUR 385). The overall sensor network set-up is simple, fast and does not require specialised training or technical knowledge.

The objectives of this study are to (1) determine the analytical performance In measuring dielectric permittivity in liquids of known ε, (2) evaluate the performance in measuring different θ in soil samples and through comparison with TDR instrumentation (3) study the data communication capabilities of the sensor network at various ranges and node sampling frequency and (4) provide recommendations for sensor deployment and how to limit the sources of error associated with drift and field installation.

## 2. Materials and Methods

### 2.1. Instrumentation

**Figure 1:**
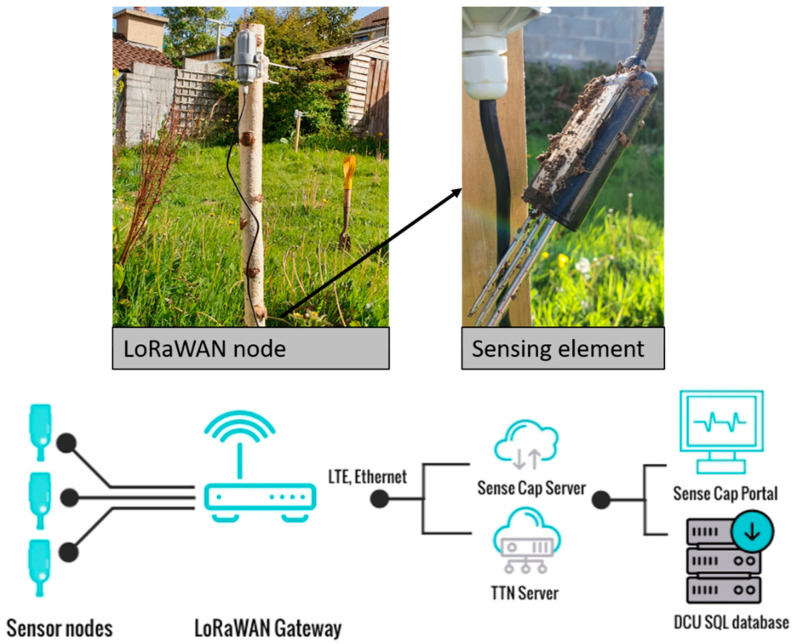
A typical sensor installation (**top**) and overall system architecture (**bottom**).

LoRaWAN Outdoor Gateway (part number 102991154) and 4× soil moisture and temperature sensors (part number 101990564) were procured from Mouser Electronic, Buckinghamshire, UK with the remaining 6 units procured from DigiKey, Ireland (Thief River Falls, MN, USA). A 4.5 dBi LoRa antenna, 868 MHz, was procured from Paradar, London, UK, while the antenna extension cable was procured from Radionics, Dublin, Ireland. The time-domain reflectrometer-based soil profiler, SoilVUE™ (parameters: ε, temperature, bulk electrical conductivity and θ) and the CR 300 data logger were procured from Campbell Scientific, Loughborough, UK. The Sense Cap node consists of the sensing element (temperature, °C and θ, %) and the sensor node controller which houses the LoRa communication module, battery and low-power microcontroller (Figure 1). The data communication architecture relies on LoRaWAN gateways to provide the coverage for data collection from the nodes and data upload to the cloud via 4G or Ethernet (Figure 1). For this study, the data were retrieved from the Sense Cap portal and further archived into an SQL database.

### 2.2. Reagents and Methods

Isopropyl alcohol (IPA) (99.5%), methanol (99.5%), acetic acid 98% (AA) and ethanol (99.5%) were all procured from Sigma Aldrich, Arklow, Ireland a subsidiary of Merck KGaA (Darmstadt, Germany).

#### 2.2.1. Intersensor Variability in Dielectric Standards

To determine intersensor variability, 10 sensor units were tested in liquid media and air of known ε. Sensors were fully immersed and allowed to collect 5 readings in each standard at 20 ± 1 °C. Apart from acetic acid, for which only one measurement was collected, all measurement were collected in triplicate.

#### 2.2.2. Soil Testing

Materials used in the experiments consisted of garden soil substrate (clay loam soil) and potting soil substrate (peat moss soil). For sample preparation, soils were air-dried for one week. Root material and fibres were removed, and the soil was sieved through a 5 mm sieve. The mixed-cell method was used to prepare sample with incremental water content. Known volumes of water were added via spraying while soil mixing was carried out using a paddle mixer. For the comparison with the TDR sensor, a bespoke sample holder of approximately 15 L volume was built (see Section 3.2) to allow testing. Packing of soil was carried out through the subsequent addition of material in layers and compaction to avoid air gaps and voids. The volumetric water content of the samples was determined using the gravimetric method and the bulk density. A soil sample ring (Ø 50 mm, height 51 mm) was used to collect fractions from the prepared samples, which were weighted before and after air-drying. The TDR sensor was positioned in the middle of the sample container, with a soil thickness around the sensor of at least 5 cm. Because the volume of influence for the TDR instrument extends 1.5–2 cm from the rods, the Sense Cap sensors were placed at least 3 cm from the rods. To limit evaporation during the experiment, the sample holder was wrapped in polyethylene film. At least 10 readings were collected from all sensors for each sample (5 min sampling frequency) by shuffling the Sense Cap sensors at various locations along the soil sample. The TDR collects data at 9 distances (i.e., 5, 10, 20, 30, 40, 50, 60, 75 and 100 cm) which were subsequently averaged to provide 1 measurement for each sample.

#### 2.2.3. Data Communication Testing

The data communication performance of the network was evaluated as a function of transmission delay and packet drop rate at different ranges and sampling frequencies. Ranges and sensor nodes are shown in Table 1, and these conditions were kept constant throughout the study. Sensors were allowed to run between 24–48 h at sampling frequencies of 5, 10, 30 and 60 min, respectively. The test site consisted of a busy urban environment with no clear line of sight between the gateway and the nodes.

## 3. Results and Discussion

### 3.1. Sensor Performance Evaluation with Known Dielectric Standards and Intersensor Variability

Although sensors with the same manufacturer part number were ordered, two slightly different probes were received. The first type of probe (nodes 3 to 10) consists of a 2.77 cm-diameter cylindrical head with three, 0.3 diameter tines protruding for 7 cm, while the second type (nodes 1 to 3) consists of a 2.7 cm-diameter cylindrical head with three, 0.4 diameter tines protruding for 5.5 cm. The main difference between the two types is the length of the tines and the response variability between probes in the same sample. In terms of the operation principle, the sensor operates similarly to the WET sensor described previously [12,20]. Briefly, the sensor returns a voltage at a fixed frequency (70 MHz). Capacitance of the material between the tines is measured, from which dielectric properties of the medium are inferred using a sensor calibration file. In the final step, measured ε is used to calculate θ according to Topp’s equation [21], where ε is the apparent dielectric permittivity of the medium and θ is the volumetric water content.

(1) $$\theta =4.3\times 10^{-6}\varepsilon^{3}-5.5\times 10^{-4}\varepsilon^{2}+2.92\times 10^{-2}\varepsilon -5.3\times 10^{-2}$$

**Figure 2:**
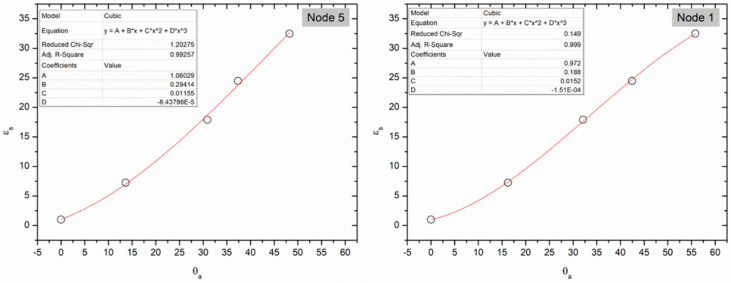
Example of unit-specific standardization equations in ε standards. Insets show equation coefficients.

To address the various soil property effects in EM-based measurements for θ, soil-specific calibrations are often recommended, although in general, suppliers provide factory-determined calibration equations. The performance of such factory calibrations has been reported in detail previously for the most common EM sensors for different soil textural classes [12]. Although multiple equations exist as linear and nonlinear, to estimate θ from sensor response [12] it is critical to minimise intersensor variability (i.e., the degree of variation in response among different sensor units) to provide reliable data from spatially distributed sensors. Permittivity is the physical property that drives the θ determination, and it is easier to provide a known permittivity using dielectric liquids than to provide a known θ in soil (i.e., due to soil heterogeneity or hydrostatic water distribution) [22]. The use of liquids with known dielectric permittivity values reduces the variability associated with solid media and provides a reproducible approach to sensor screening. Liquids are “ideal” dielectric media because of their well-defined properties which overcome complications associated with the use of soil such as air gaps near conductors and density variations [22]. For this purpose, a range of solvent types and mixtures were selected to cover the ε range (Table 2). The SenseCap sensors used only output the temperature and θ (%) data, while the ε data are only available through the serial connection. Equation (1) (used by the manufacturer) was utilised to solve for ε from θ. The average ε presented in Table 2 shows a good correlation between the ε_s_ (standard dielectric permittivity) and ε_a_ (measured dielectric permittivity) for the low range (1–24.5) for all the nodes. A significant shift is noticed starting with methanol, where all the sensors overestimate the ε_s_. A similar effect has been reported before for the 10HS sensor with slight overestimation in the 0–37 ε range [23]. In this case, the error is larger, which suggests that the calibration file used is not ideal. It is possible that calibration was achieved using a two-point calibration (i.e., in air and water). Standardising the sensor response to ε_s_ offers two key advantages: it reduces the intersensor variability and converts the sensor response to a more accurate ε which in turn can be used for more reliable θ calculations. Standardisation can be achieved by converting the sensor output θ_a_ (%) to ε_a_ and finding the equation between ε_a_ and ε_s_ or by finding the equation between θ_a_ (%) and ε_s_. The later approach was used as it was found to provide better root-mean-square error values (RMSE) for the 1–32.5 range and R^2^ > 0.98 for all nodes. Unit-specific standardisation equations in dielectric permittivity standards were developed for each node and two example are provided in Figure 2.

**Figure 3:**
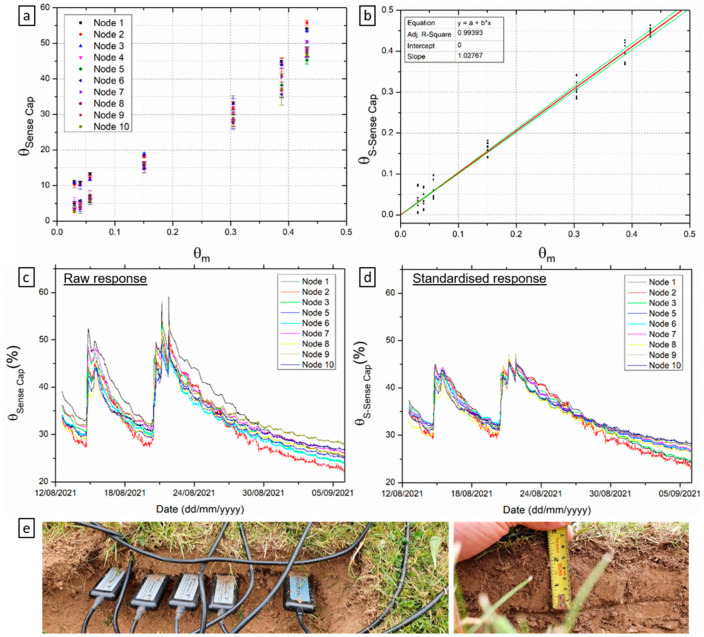
Raw and standardized response from the Sense Cap units. (**a**) raw sensor response with increasing θ_m_ in prepared soil samples, error bars represent the standard deviation of at least 9 measurements, 3 measurements at 3 different positions in the sample (**b**) standardised sensor response to θ and linear relationship between actual and estimated θ; (**c**) raw response time-series data; (**d**) standardised response time-series data; (**e**) experimental set-up for data in (**c**) showing sensor installation within close proximity to each other, at approximately 5 cm depth.

It was found that the shorter probes (nodes 1–3) produced consistently higher readings than the longer probes (nodes 4–10), as shown in Table 2. Applying the standardization equations was found to reduce the intersensor variability. An example is shown in Figure 3a,b for a set of samples prepared by incremental addition of water, where inter-sensor variability is reduced considerably at the extremities of the θ range. For these experiments, Equation (1) was used to compute the standardised θ from ε_s_. In addition, the standardization corrects for the overestimation of ε_s_ and the standardised θ (θ_S-Sense Cap_, Figure 3b) values are much closer to the measured θ (R^2^ = 0.99, slope = 1.027). Another example to support the response standardisation in shown in Figure 3c,d as time series for 4 weeks of data, with all 10 sensors deployed at approximately 5 cm below the root line. Note the Y axis are identical in both panels to facilitate direct comparison of raw and standardised data. As before, the intersensor variability and θ are reduced.

Furthermore, data collected in Table 2 are essential to providing quality control (QC) for when sensors are operating in situ. Sensor drift due to corrosion, or hardware issues, can cause sensor response to deviate from the real value. Often, this response drift is overlooked, and ‘bad’ data can be taken as reliable, unless QC measures are in place. Identifying sensor nodes that are malfunctioning at an early stage and discarding or correcting the associated data is good practice and cost effective.

### 3.2. Cross-Comparison with TDR Sensor for Varying θ

**Figure 4:**
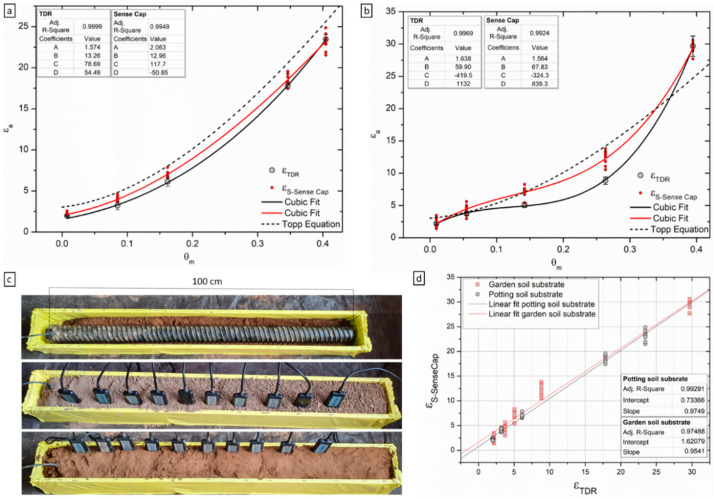
Comparison of Sense Cap sensors with the TDR instrument; relationship between the actual θ_m_ and the ε_a_ measured with the Sense Cap and TDR sensors (see legend) in garden soil substrate (**a**) and potting soil substrate (**b**); error bars on the ε_TDR_ represent the standard deviation of at least 45 readings (5 readings/depth, 9 depths along the probe), ε_SenseCap_ is the average of 5 readings/unit (**c**) experimental set-up showing the soil sample holder and sensor placement in the same sample—example provided for garden soil substrate; (**d**) linear relationship between standardized ε_SenseCap_ and ε_TDR_ (see figure insets).

There are two common methods used for laboratory and field calibration of soil moisture sensors [23,26]. The more commonly used method, the mixed-cell method or the disturbed calibration method, uses measurements made in cells containing soil mixed with different known amounts of water to provide distinct points describing the relationship between the ε and θ [26]. The second method is known as the undisturbed calibration method, or the infiltration-addition method, and was described previously [27]. The main difference between the two is the soil structure which is removed when using the first method through soil sieving, grinding and subsequent mixing with water. Most sensor manufacturers recommend that calibration is undertaken on soil in which the structure has been removed, although it is argued that ideally the structure should be maintained to limit uncertainty associated with the pore size distribution and the small volume of influence of some probes. Due to the size of the TDR instrument used (Figure 4c), the mixed-cell method was used in this study although it is in general more laborious and results in variable bulk densities. Two types of soil substrates were used for the cross-comparison study: garden soil substrate (clay loam soil) and potting soil substrate (peat moss soil). None of the soils tested showed a good fit with the Topp’s equation, although polynomial third equation models could be fitted to the experimental data to provide R^2^ > 0.99 (Figure 4a,b; see insets for coefficients). Such soil-specific calibration curves are generally developed for field application with soil samples from the site, and it has been shown previously that both these types of soils tend produce high RMSD values when fitted with the Topp equation. For example, results are in agreement with previous results on clay and rockwool [23]. The potting soil substrate (peat moss soil) used here can be classified as organic soil, for which the response seems to be best described by Schapp’s equation for organic forest soils [28] with a similar response reported previously [12]. The offset from Topp’s equation for these types of soils is driven by the lower density and higher porosity of the solid phase [29]. The garden soil substrate used can be classified as clay-rich soil, for which deviation from Topp’s equation were reported before with increasing clay content [12,20]. It is considered that this deviation is due to the particle shape, clay mineralogy and high surface area (bound water) which in turn alter the ε_a_ [12]. Another reason proposed is the nonrigid structure of many clay minerals and their ability to shrink and swell, which could maintain connectivity between interaggregate pores at low water contents [30]. In turn, this effect produces lower observed ε at low θ and higher ε at high θ in relation to the Topp’s function, as noticed here (Figure 4a).

In this experiment, the main aim was to compare the low-cost sensor’s response as dielectric permittivity with the TDR instrument. For both soils, the Sense Cap probes overestimate ε by comparison with the ε_TDR_ (Figure 4a,b,d). This offset is minimal in the potting soil substrate, with slightly higher ε_SenseCap_ throughout the tested range but in good agreement with the ε_TDR_. On the other hand, there is a significant overestimation in the garden soil substrate, particularly the low–middle range. These differences can be attributed to differences in the measurement frequency and operation mode (capacitance vs. TDR) [12], or variations in sensor characteristics including probe geometry, printed circuit board design, and sensor head sensitivity [31]. Additionally, observed differences could be a soil packing artefact where the lower ε_TDR_ in the middle region is due to the presence of more air pockets. Since the TDR measurements were collected as an average of data coming from all the rods along the profiler and from a much higher volume of influence, it is possible more air pockets are present. It is worth noting, the results are consistent with the response of Wet2, a similar sensor in design and operation mode, where previous studies report an overestimation of ε when compared to the TDR instrumentation [12,32,33].

### 3.3. Sources of Errors

**Figure 5:**
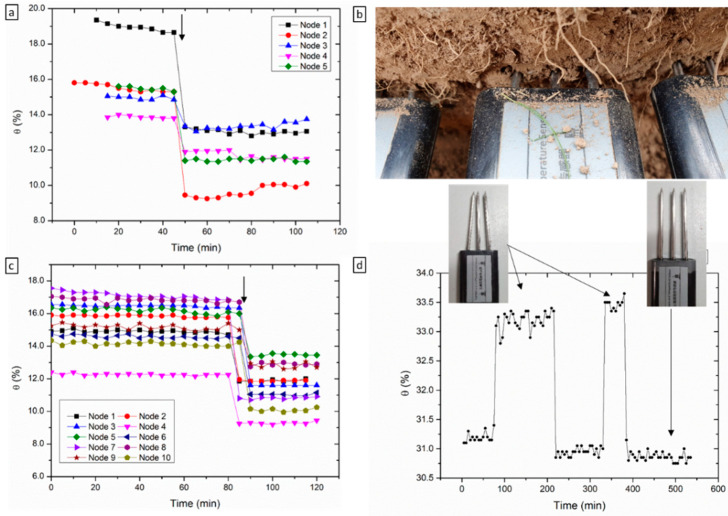
Errors and sources of errors associated with sensor installation. (**a**) Data showing sensor response before and after partial exposure to air—example provided in (**b**); (**c**) data showing sensor response before and after the presence of an air interface between the probe and soil—introduced through repeated shaking; (**d**) data drift associated with tines deflection.

**Figure 6:**
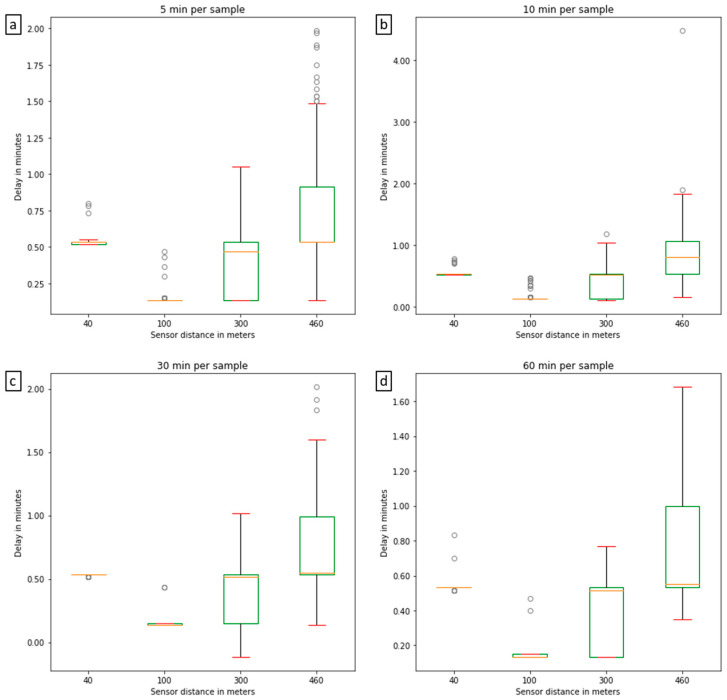
Boxplots of data transmission delay (**a**) 5 min sampling rate, N = 485; (**b**) 10 min sampling rate, N = 825; (**c**) 30 min sampling rate N = 86 and (**d**) 60 min sample rate N = 49; whiskers are calculated as 1.5 times the IQR (interquartile range) and the circles represent the outliers.

The accuracy and precision of the sensor data is dependent on sensor performance and sensor installation. Custom-designed sensor deployment tools are usually provided by high-end sensor manufacturers to reduce user errors. For low-cost sensors however, such tools are not provided. Furthermore, in most cases, no sensor deployment recommendations or guidelines are given. In this context, a series of installation configurations were investigated to determine sensor response variability and to provide sensor installation guidelines. Measurement errors were observed particularly when the tines of the probe are partially exposed to air or air gaps are present. This can happen when the probe is not fully pushed into the soil, and given that the dielectric permittivity is a function of the volume influence, lower readings are observed (Figure 5c). Another example which is more common and requires considerable attention is the insertion of an air gap between the probe and the soil through probe disturbance (Figure 5b). This can happen immediately or during probe installation and can be caused by accidentally moving the probe from its original position or after covering of the probe with soil. Tines’ deflection, caused by very dry compacted soil and tines angular off-set has also been observed. Figure 6d shows an example of data associated with this error in ethanol. Upon installation, it is not possible to know if the tines are parallel or angled, which reduces the volume of influence of the probe and causes an increase in ε. A good approach to minimise this is to check and align probe tines to be parallel and/or to use a tool for piloting the holes. 

### 3.4. LoRaWAN Performance

LoRaWAN technology is well-known for its long-range data acquisition with low power consumption, however LoRaWAN has limited messaging capabilities which may cause transmission delay or even data loss in the network. Therefore, it is useful to evaluate the data communication capability of the sensor network. Various settings in the evaluation can have an effect on the data transmission and the Packet Error Rates (PERs) of the network. A high PER will consequently increase the duration of data transmission. Therefore, by analysing the transmission time between the sampling time (at individual nodes) and the data collection time (i.e., the time at which the gateway receives the data), it is possible to determine the delay. The delay includes uplink (time on air) and the default time offsets for receiving the frame on the gateway side [34]. Since the time stamp associated with the individual nodes collecting a measurement is not available, it was estimated using the sampling frequency at the node and the *t* = 0 (i.e., the time stamp at which the nodes collect the first measurement with the new sampling frequency). Using the newly estimated sampling times and the time difference between two adjacent samples it was possible to calculate the delay (Figure 6). The delay medians for the four sampling frequencies are 31 s, 31 s, 32 s and 32 s, respectively. Thus, there is no observed significant difference in data transmission delay among different sampling rates. However, as shown in Figure 6, there is an increase in the median delay with distance from the gateway, independent of the sampling frequency. For example, in the “30 min per sample” subplot, the IQRs of delays for the distances from 40 m to 460 m are 0, 0.017, 0.383 and 0.458 min, respectively, while the medians for the distance 100 m, 300 m and 460 m are 0.133, 0.517 and 0.55 min. This observation is a consequence of the Adaptive Data Rate (ADT) scheme used in LoRaWAN, which aims to minimise energy consumption and maximize throughput by adjusting the data rate for every end node. ADR controls the transmission parameters, namely Bandwidth (BW), Spreading Factor (SF), Transmission Power (TP) and Coding. Rate (CR) [35]. ADR changes the data rate based on simple rules. For example, if the link budget is high, the data rate can be increased by increasing the SF, while if the link budget is low, the data rate can be lowered by decreasing the SF [36]. The sensors tested operate at an SF between 7–12, and it is known that large SFs allow for a longer communication range while increasing the time on-air and consequently the off-period duration [36]. Accordingly, there is trade-off between SF and transmission range, with lower delays and lower SFs present for shorter ranges [36]. The higher delay for the nodes positioned at 40 m nodes was caused by the positioning in relation to the gateway. The sensor nodes at the other distances were in the antenna field of view (i.e., facing the antenna) while the nodes at 40 m were positioned behind it. This was later confirmed to be the cause, by positioning the nodes in the same field of view.

In addition to the four ranges presented here, a fifth range of approximately 740 m was originally used in the experimental design. However, this range was excluded from the analysis as more than 50% of the data packets were lost. According to the manufacturer’s specifications, the antenna should provide a range of up to 2 km with no clear line of sight and up to 10 km with a clear line sight, which is not substantiated by these results. This finding prompted the replacement of the included Sense Cap antenna (Antenna A) with a 4.5 dBi LoRa antenna, 868 MHz, from Paradar, UK (Antenna B). An initial investigation revealed promising results for Antenna B with a range of approximately 1.5 km and with no obvious data loss. As a cross-comparison for data drop rate, the tests carried out with Antenna A were reproduced over a period of 12 days with Antenna B, maintaining the same distances from the gateway. Given fixed data sampling frequencies, the total number of packages can be estimated and the drop rate can be calculated as:

(2) $$drop\;rate=\frac{\mathrm{total}\;\mathrm{number}\;\mathrm{of}\;\mathrm{packages}-\mathrm{number}\;\mathrm{of}\;\mathrm{received}\;\mathrm{packages}}{\mathrm{total}\;\mathrm{number}\;\mathrm{of}\;\mathrm{packages}}$$

It was found there is no significant difference between the two antennas for the range tested and that the drop rate increases with range (Table 3). While Antenna A had a smaller drop rate at shorter ranges it shows a higher drop rate at higher ones. The % drop rate for the two antennae is consistent with previous reports when LoRaWAN is used for environmental applications [37]. Data loss through operational issued or sensor drift can have a deleterious effect on the overall sensor network performance and is not desirable. Two options are available to mitigate data loss: increase the overall data transmission performance or use imputation algorithms to introduce missing values and maintain the sample size [37].

## 4. Conclusions

This study demonstrates a low-cost soil moisture sensor network based on laboratory and field measurements with dielectric permittivity standards and soil media. The embedded sensor calibration, in-built within each sensor node, does not accurately predict the ε of liquid standards which consequently leads to inaccurate θ estimations. Namely, two shortcomings were identified: the sensors overestimate the ε_s_ particularly for values > 32, and a high intersensor variability is present between the two sensor types tested. To normalise the sensor output, the raw response for each unit was standardised to the ε_s_, through unit-specific equations. This approach was found to reduce the intersensor variability and provide robust estimates of θ in soil samples with known θ. Furthermore, when the sensor was tested against a TDR instrument, the two probes were found to be in good agreement throughout the tested range. Although the ε was overestimated for the low–middle θ range for the heavy clay soil, this seems to be consistent with similar sensors reported in the literature. Sensor drift due to corrosion, or hardware/electronic issues, can cause sensor response to deviate from the ground truth. Identifying sensor nodes that are malfunctioning at an early stage is essential for the collection of robust data. The collected data on liquid standards provide the baseline for QC measurements while sensors are deployed. Sources of errors associated with suboptimal probe installation were identified and discussed. Namely, measurement errors were observed when the tines of the probes were partially exposed to air or air gaps and when the tines were deflected from their parallel configuration upon installation. The data communication performance of the network was evaluated in terms of packet drop rate at different ranges and sampling frequencies. It was noticed that the drop rate increased with the distance from the gateway, while sampling frequency had no effect. The range provided by the Sense Cap antenna was found to be small (approximately 500 m) and was significantly improved by upgrading to a 4.5 dBi LoRa Paradar antenna (approximately 1500 m).

In summary, the Sense Cap soil moisture sensor network evaluated in this study shows potential for in situ implementation for soil moisture monitoring. The off-the-shelf all-in-one solution provided is low-cost and user-friendly (easy and fast installation which does not require specialised training). Standardisation of sensor units is advised to achieve robust estimates of θ and improve the analytical performance. Considering all of the above, the optimal set-up for efficient, accurate and reliable soil moisture networks that can provide both spatial and temporal resolution should be hybrid and encompass multiple low-cost nodes accompanied by at least one TDR profiler for validation purposes.

## Figures and Tables

**Table 1:** Distance from the gateway for each group of sensor nodes.

Distance from Gateway (m)	40	100	300	460
Node ID	1, 2	3, 4	5, 6	7, 8

**Table 2:** Measured ε_a_ and intersensor variability for 10 units.

Media	ε_s_ (F m^−1^)	ε_a_ (F m^−1^)									
		Node 1	Node 2	Node 3	Node 4	Node 5	Node 6	Node 7	Node 8	Node 9	Node 10
Air	1	1.88	1.88	1.88	1.88	1.88	1.88	1.88	1.88	1.88	1.88
AA	7.25 *	8.71	8.11	8.23	7.07	7.48	6.56	7.37	7.58	7.44	7.32
IPA	17.9	18.10 ± 0.06	16.19 ± 0.21	16.85 ± 0.144	18.10 ± 0.22	17.22 ± 0.11	16.84 ± 1.01	18.26 ± 0.55	18.11 ± 0.52	18.51 ± 0.08	17.18 ± 0.67
Ethanol	24.5	27.62 ± 0.05	23.80 ± 0.18	24.69 ± 0.49	24.70 ± 0.90	22.41 ± 0.05	22.95 ± 1.45	24.45 ± 0.43	24.20 ± 0.93	25.88 ± 0.46	24.81 ± 2.08
Methanol	32.5	48.17 ± 0.22	46.68 ± 0.39	48.59 ± 0.44	43.41 ± 0.10	35.42 ± 1.38	39.11 ± 0.19	41.94 ± 0.30	40.66 ± 0.25	38.61 ± 1.15	42.44 ± 1.11
Mix 1 **	42.99 **	67.07 ± 1.44	62.77 ± 1.61	65.04 ± 1.63	53.01 ± 1.14	47.66 ± 1.02	49.96 ± 0.99	51.35 ± 1.37	51.16 ± 1.16	52.91 ± 0.13	50.54 ± 0.25
Mix 2 **	60.9 **	78.61 ± 1.11	76.98 ± 1.84	76.88 ± 1.88	77.87 ± 0.23	75.50 ± 1.31	80.25 ± 2.40	77.61 ± 1.34	79.02 ± 0.79	78.60 ± 1.55	75.58 ± 2.58
H_2_O	80.1	81.36 ± 0.05	81.30 ± 0.29	81.39 ± 0.06	81.14 ± 0.01	81.14 ± 0.25	81.17 ± 0.03	81.15 ± 0.02	81.12 ± 0.04	81.17 ± 0.07	81.12 ± 0.02

AA—Acetic acid (98%). IPA—Isopropyl alcohol. ε_s_—dielectric permittivity of standards, at 20 °C. ε_a_—apparent dielectric permittivity, measured by the sensors and retrieved using Equation (1); averages reported for triplicate measurements with 5 readings/replicate with the exception of AA where 3 readings were collected. * Dielectric permittivity of AA (98%) was obtained from [24]. ** Mixture 1 and Mixture 2 were prepared from volumetric ratios of water and ethanol from [25].

**Table 3:** Communication data drop rate.

Range	40 m	100 m	300 m	460 m
Antenna A	5.48%	1.15%	3.83%	13.64%
Antenna B	6.81%	1.91%	4.91%	9.69%
N_antenna A_	1569	1560	1565	1547
N_antenna B_	2510	2511	2506	2509

N—the total number of packages (the maximum number of packages calculated for each node from the sampling frequency and time stamps).

## Data Availability

The datasets supporting the conclusions of this article are included within the article. Raw data files are available on request from the authors.
